# A Multi-Sensing Technology Approach for the Environmental Monitoring of the Ystwyth River

**DOI:** 10.3390/s25216743

**Published:** 2025-11-04

**Authors:** Edore Akpokodje, Nnamdi Valbosco Ugwuoke, Mari Davies, Syeda Fizzah Jilani, Maria de la Puera Fernández, Lucy Thompson, Elizabeth Hart

**Affiliations:** 1 Department of Computer Science, Aberystwyth University, Aberystwyth SY23 3DB, UK; nnu1@aber.ac.uk; 2 Department of Life Sciences, Aberystwyth University, Aberystwyth SY23 3DA, UK; mwd9@aber.ac.uk (M.D.); elh18@aber.ac.uk (E.H.); 3 Department of Physics, Aberystwyth University, Aberystwyth SY23 3BZ, UK; sfj7@aber.ac.uk; 4 Institute of Biology, Environmental and Rural Sciences, Aberystwyth University, Aberystwyth SY23 3EE, UK; mad79@aber.ac.uk; 5 Department of English and Creative Writing, Aberystwyth University, Aberystwyth SY23 3DY, UK; let22@aber.ac.uk

**Keywords:** water quality monitoring, Ystwyth River, sensors, interactive Web-Based map, environmental surveillance, real-time data

## Abstract

**Highlights:**

**What are the main findings?**

- Real-time environmental surveillance of the Ystwyth River was conducted using AquaSonde sensors (Aquaread Water Monitoring Instruments, Kent, United Kingdom).
- High-frequency monitoring captured short-term turbidity and nutrient fluctuations linked to rainfall and agricultural activity.
- Land-use analysis showed improved grassland and livestock farming as major influences on water-quality variability.
- A web and mobile application was developed for data visualisation with site-specific pollutant markers.

**What are the implications of the main findings?**

- Continuous sensor monitoring improves temporal resolution and enables real-time detection of event-driven pollution.
- Integrating sensor data with land-use mapping supports identification of pollution hotspots and informed catchment management.
- The system promotes proactive water management, with future AI and satellite integration to enhance prediction and mitigation.

**Abstract:**

Monitoring water quality in Welsh rivers has become a critical public concern, particularly in efforts to address pollution and protect the environment. This study presents the development and assessment of an interactive web and mobile application, featuring a real-time mapping interface built using the Mapbox framework. The platform provides stakeholders, including farmers, environmental agencies, and the public, with easy access to real-time water quality data using the Ystwyth River in Mid-Wales as a trial system. Users can click on map markers to view sensor readings for key water quality parameters. These include pH, electrical conductivity (EC), temperature, dissolved oxygen (DO), total dissolved solids (TDS) and nutrients levels such as nitrate (NO_3_). This paper focuses on the feasibility of combining in situ sensor technology with a user-friendly mobile app to enable stakeholders to visualize the impact of land management practices and make informed decisions. The system aims to enhance environmental surveillance, increase transparency, and promote sustainable agricultural practices by providing critical water quality information in an accessible format. Future developments will explore the integration of artificial intelligence (AI) for predictive modelling and satellite data for broader spatial coverage, with the goal of scaling up the system to other catchments and improving proactive water quality management.

## 1. Introduction

There is an increasing need for effective management of river water quality inland. This has underscored the importance of real-time, rapid, and continuous monitoring, particularly in addressing the challenges posed by river eutrophication. Excess phosphorus and nitrogen contribute to eutrophication, resulting in a decline in water quality and adversely affecting aquatic biodiversity [1,2]. Approximately 90% of the land area in Wales is used for agricultural purposes [3], and studies in North Wales have shown that intensive farming in high-quality agricultural areas contributes to elevated nitrates, phosphorus, and bacterial coliform in rivers [4,5,6]. Runoff from livestock defecating near streams, influenced by stocking density and soil compaction, can introduce phosphorus and nitrogen into the water [7]. Additionally, silage production on lower pastures often requires the application of inorganic fertilizers, which are prone to leaching during rainfall events, particularly on clay soils [8]. These agricultural practices increase the risk of eutrophication, compounding the effects of other land covers such as historic mines and coniferous forests [5]. To mitigate these impacts, strategies include implementing riparian buffers, where vegetation near the stream filters nutrients before they enter the water [9], and adopting controlled grazing strategies, such as restricting grazing during winter in saturated areas near waterways [7]. River quality in Wales remains under ongoing scrutiny, with only about 44.5% of rivers currently meeting good ecological status, highlighting the need for further improvements in water quality across the region [10].

The novelty of this study lies in the first application of high-frequency, multiparameter in situ sensors on the Ystwyth River combined with the development of an interactive web and mobile application for real-time data visualization. Unlike previous monitoring approaches that relied on periodic sampling, the proposed system provides continuous, accessible, and transparent information on key water quality parameters. By integrating environmental data with a stakeholder-focused digital platform, the work establishes a prototype decision-support tool for farmers, regulators, and the public. Furthermore, the study introduces a scalable framework that can be extended through artificial intelligence and satellite integration, positioning the system as a novel contribution to proactive and policy-relevant river basin management.

Given the crucial role land use plays in water quality [11], addressing these issues is especially important in rural Wales, where agricultural practices and environmental conservation intersect. The Wales Better River Quality Taskforce has emphasized the importance of real-time surveillance to address these challenges, especially for managing CSO discharges and agricultural pollutants. The Water Resources (Control of Agricultural Pollution) Regulations introduced in Wales in 2021 specifically target nitrate pollution from agricultural sources. These regulations complement River Basin Management Plans (RBMPs) developed under the EU Water Framework Directive (WFD), which aim to mitigate pollution in river basins across the country. This ensures that Welsh rivers meet environmental objectives for good water quality, compliance [6]. Real-time monitoring enables rapid responses to emerging issues, facilitating proactive water management, ensuring compliance with environmental standards, and helping to preserve river ecosystems [10,12].

To streamline data collection for water quality monitoring and test a system for delivering real-time data visualization, an AquaSonde sensor was installed in the Ystwyth River for environmental monitoring. The Ystwyth River is a 25-mile-long river that flows through Ceredigion, mid-Wales and faces significant risks of eutrophication due to multiple sources of nutrient pollution. The river originates in the Cambrian Mountains, where several tributaries, notably the River Diliw, flow from the western slopes of Pumlumon, situated on the border between Ceredigion and Powys. The catchment area of 76 square miles encompasses diverse land covers, including coniferous forests, woodlands, historic mines (silver, lead, and zinc), and agricultural activities such as mixed livestock farming and silage production [13]. This test case focused on developing an interactive map and app that has the potential to provide stakeholders with real-time water quality data. Traditional water quality monitoring methods, which rely on on-site measurements, sample collection, and laboratory analysis, are resource-intensive in terms of labour, materials, and costs [14]. In contrast, real-time monitoring offers a more efficient and immediate means of assessing water quality, allowing stakeholders such as environmental agencies, local authorities, farmers, and conservation groups to respond more quickly to potential environmental threats. By addressing these issues, farmers can enhance both the economic and environmental sustainability of their enterprises. Additionally, this approach helps prepare them for evolving governmental regulations, such as the Sustainable Farming Scheme commencing in 2026 [15].

Recent work on the River Thames and the Essex Colne River in the United Kingdom has demonstrated the potential of combining cloud-based systems with novel biosensor technologies to deliver real-time, publicly accessible water quality forecasts [16]. Such systems are increasingly valuable as short-term impairments—including cyanobacterial blooms, sediment plumes, and acute pollution incidents—pose growing risks to water resources and public health. The Thames and Colne are particularly important, serving as major water supply sources for London and South-East England and supporting extensive recreational use. Beyond forecasting, the system was also capable of estimating toxicity and integrating citizen science observations through the AquaScope platform (www.aquascope.com) URL accessed on 6 October 2025, which provides an accessible interface for data synthesis and dissemination. This line of research highlights the broader trend toward leveraging digital platforms and participatory monitoring approaches in water quality management. An approach that parallels this study of the Ystwyth River, where a mobile application is used to display sensor-derived pollution indicators.

This test case demonstrates how real-time data collection and visualization tools can provide continuous, high-resolution information on various water quality parameters. This is particularly important in Wales, where rivers are highly susceptible to pollution from both agricultural runoff and urban development [6,17]. By offering real-time insights through an interactive map, this system empowers stakeholders to manage and mitigate nutrient pollution more effectively and ensure regulatory compliance. Ultimately, this approach supports the sustainable management of rural river systems and the preservation of biodiversity within Wales.

To situate this work within the wider context of recent research on IoT and environmental monitoring, the section below provides a detailed review of relevant studies and outlines how this approach differs from existing platforms.

### State of the Art and Positioning of the Work

Recent developments in water quality monitoring demonstrate rapid progress in three complementary areas: (i) low-power Internet of Things (IoT) sensor networks for in situ measurement, (ii) edge-cloud architectures for real-time analytics, and (iii) remote-sensing technologies for large-scale spatial assessment. Low-cost LoRa- and LoRaWAN-based systems have achieved practical, real-time measurements of pH, EC, temperature, and turbidity, using Message Queuing Telemetry Transport (MQTT) or cloud-based dashboards to transmit data from rural monitoring sites [18,19,20]. Edge-cloud frameworks have further reduced latency and bandwidth demands by performing preliminary analysis locally, allowing rapid anomaly detection and efficient data transmission to the cloud [21]. On a larger spatial scale, the integration of biosensors with dynamic catchment models has enabled real-time water quality forecasting, as shown in recent applications to the Thames and Colne Rivers [16]. Additionally, remote-sensing innovations such as adaptive high-speed echo data acquisition for bathymetric LiDAR now provide high-resolution, three-dimensional observations of river morphology and flow dynamics [22]. These technologies, together with broader reviews on IoT-machine learning integration for environmental monitoring [23,24], provide the technological foundation for modern river surveillance systems.

The approach presented in this study differs from previous research in several key aspects. First, it emphasizes stakeholder-facing usability rather than purely technical feasibility. Many IoT and edge-cloud systems have proven telemetry or model accuracy, yet few have produced a publicly accessible, user-friendly interface tailored to farmers and regulators in specific catchments. Second, it delivers continuous, high-frequency in situ sensing linked directly to land management decisions. While prior studies have validated communication efficiency and predictive modelling, this work captures high-resolution data (15 min intervals) and visualizes it in real time to support adaptive farm practices and targeted regulatory inspections. Third, the proposed platform bridges in situ sensing and remote-sensing modalities, positioning ground-based sensors as both validation tools and triggers for broader satellite or aerial data integration. Finally, the paper outlines a scalable roadmap integrating artificial intelligence (AI) and satellite data to extend spatial and temporal coverage, aligning the platform with proactive, policy-relevant water resource management.

In summary, compared with LoRaWAN prototypes, edge–cloud frameworks, and satellite or LiDAR-based forecasting systems, the novelty of this study lies in the operational coupling of high-frequency AquaSonde monitoring with a stakeholder-centred digital interface. This dual focus on technological capability and end-user accessibility provides a new model for transparent, real-time, and sustainable river basin management in the Welsh context. As discussed later in the Section 1 and Section 3, this research complements and extends existing IoT and remote-sensing approaches by focusing on end-user interaction and regional environmental policy needs.

## 2. Materials and Methods

### 2.1. Study Area

**Figure 1:**
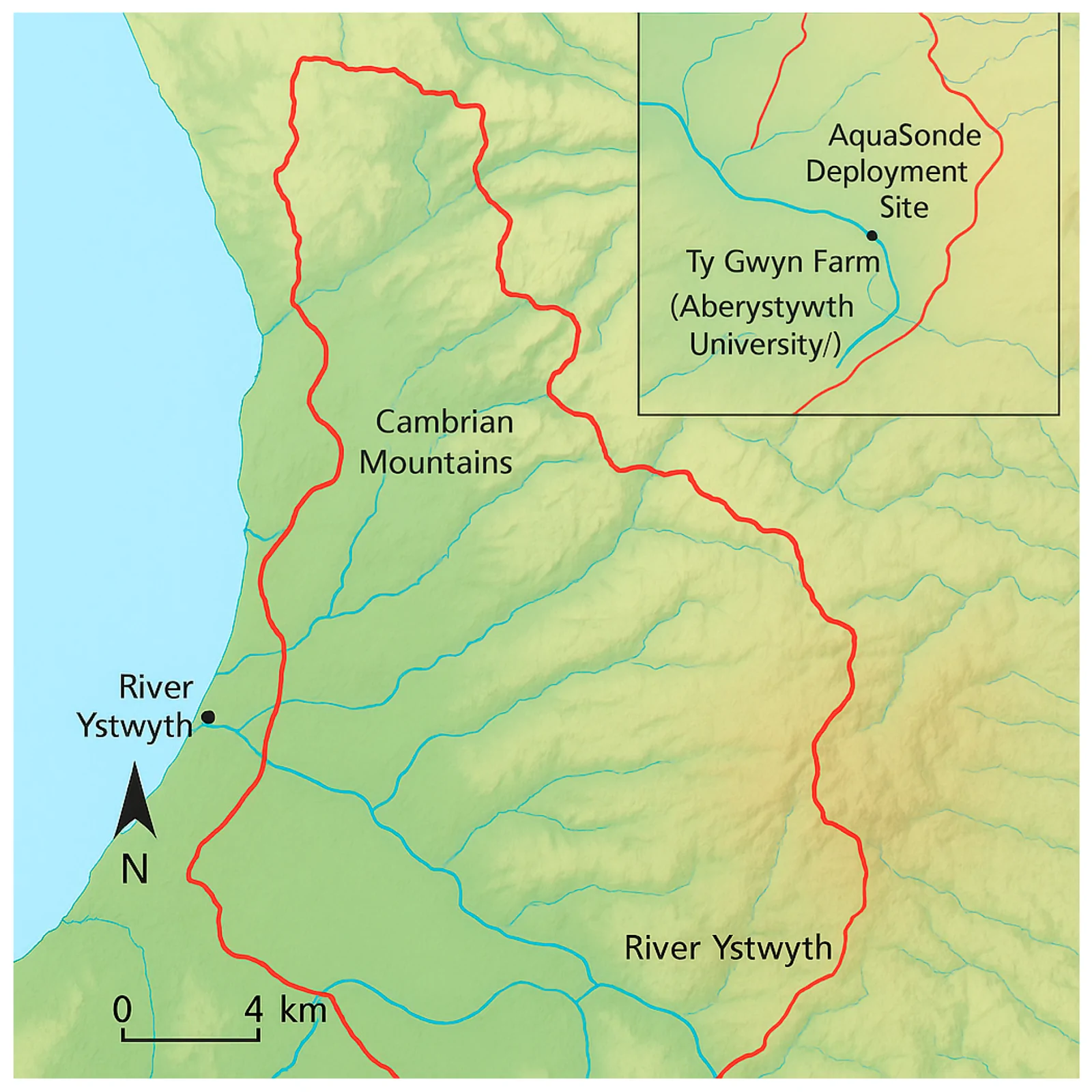
Map showing the catchment area for the Ystwyth River (outlined in red), from the Cambrian Mountains to Aberystwyth. The inset highlights the location of the AquaSonde deployment site and its proximity to the tributary Nant Pant-yr-haidd, and Ty Gwyn farm (Aberystwyth University).

The AquaSonde was deployed for a period of two months (May to end of June) to test proof of concept. It was situated in the Ystwyth River, just downstream from the adjoining tributary Nant Pant-yr-haidd, which flows past Aberystwyth University’s Ty Gwyn farm (Figure 1). The River Ystwyth flows through varied topography within a 193 km^2^ catchment area, extending from the Cambrian Mountains to Cardigan Bay at Aberystwyth Harbour. Hydrographs indicate that the upper catchment is characterized by flashy hydrology, meaning flow can fluctuate both frequently and rapidly [25], and has short lag times under two and a half hours with significant seasonal variability [26]. Tributaries such as Nant Pant-yr-haidd flow through adjacent improved grasslands in the lower river valley, contributing water and potentially agricultural runoff.

**Figure 2:**
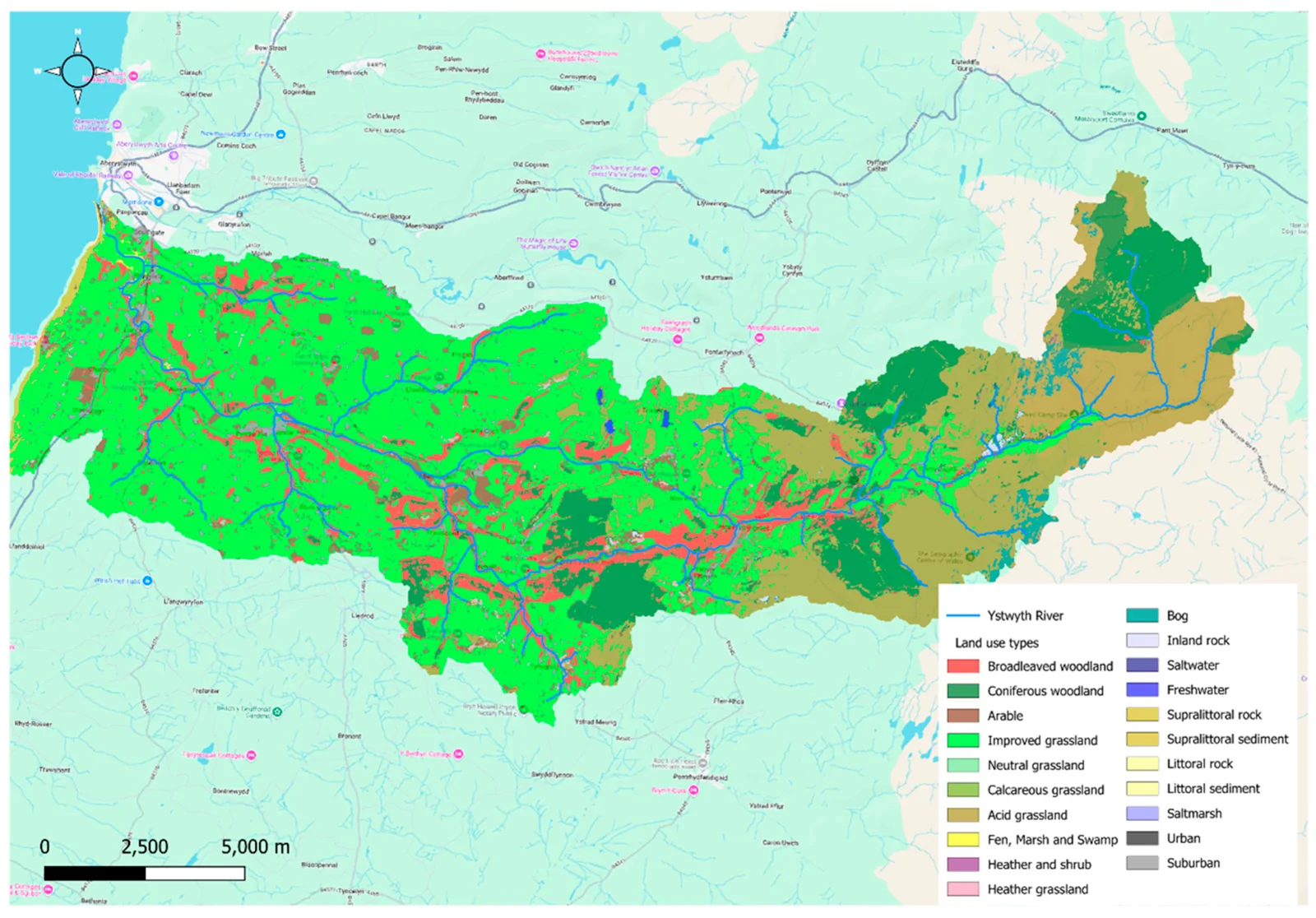
Land use types within the Ystwyth River catchment as defined by [28], highlighting the habitats and activities that may influence water quality. The AquaSonde was located within the lowland improved grassland area used for livestock farming.

The upper river valley consists predominantly of peat bogs, unimproved grassland, and semi-improved grassland used for sheep grazing. The surrounding valley sides are covered by extensive coniferous plantations, which contribute to organic materials and may cause acidification in upstream sections [27]. In the lower valley plain, there is a mixture of livestock activities on improved grasslands (Figure 2). Here, the application of fertilizers and slurry, combined with higher stocking densities and mechanical operations like silage cultivation, may lead to nutrient loss and increased sediment loads entering the River Ystwyth and its tributaries.

The probe deployment site was selected due to its proximity to the tributary, which flows directly through a farming enterprise on improved grassland, and for its ease of access. Weather conditions, including rainfall and temperature, were recorded throughout the deployment period to contextualize variations in the collected data.

### 2.2. Sensor Selection

Selecting the appropriate sensors was essential for accurate and reliable data collection. Sensors needed to be durable, dependable, and capable of operating effectively in the specific environmental conditions of the River Ystwyth. The primary sensor types considered for this study include:

- Multiparameter sondes: Devices can simultaneously measure various parameters, including pH, dissolved oxygen (DO), temperature, electrical conductivity (EC), and turbidity.
- Nutrient sensors: Specialized sensors designed to detect concentrations of nitrates and phosphates.
- To effectively monitor the water quality of the River Ystwyth, the following key parameters were identified:
- Temperature: Influences DO levels and overall aquatic life.
- pH: Indicates the acidity or alkalinity of the water, affecting chemical solubility and biological availability.
- Dissolved Oxygen (DO): Essential for the survival of aquatic organisms.
- Electrical Conductivity (EC): Reflects the amount of dissolved salts and other chemicals.
- Turbidity: Measures water clarity, which can be affected by suspended particles.
- Nutrient levels: Including nitrates (NO_3_) and ammonia, which can contribute to eutrophication when present in excess.

**Figure 3:**
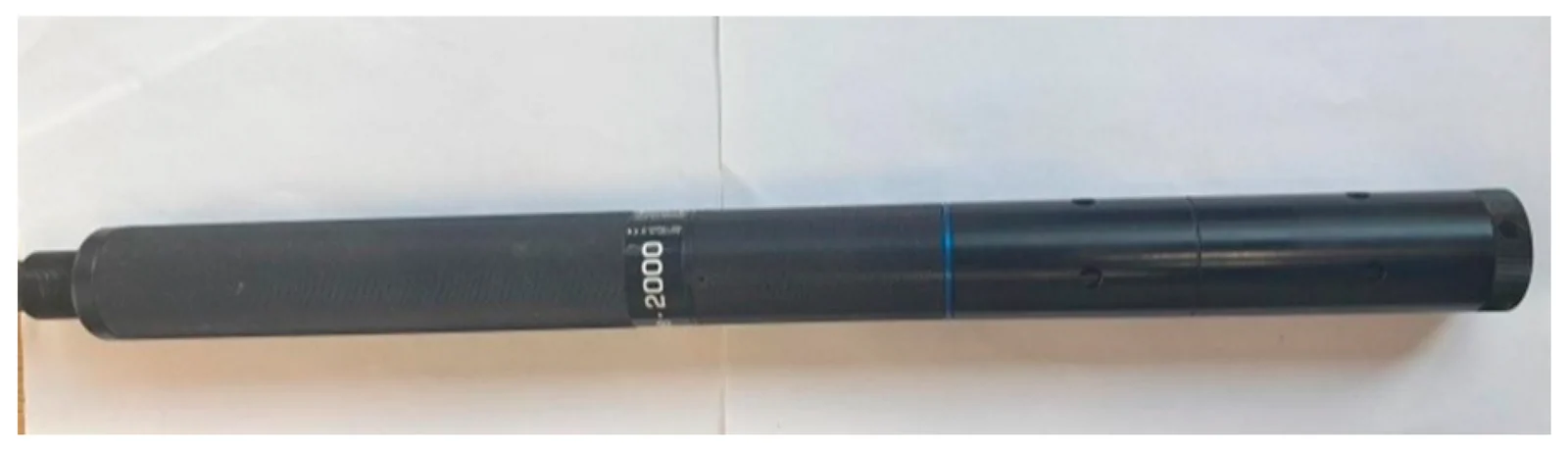
The AquaSond-2000 (AS-2000) multiparameter water quality monitoring sonde used for continuous monitoring during the study period.

A multiparameter sonde (Figure 3), the AquaSond-2000 water quality monitoring sonde (AS-2000), was selected for continuous water quality monitoring. The AS-2000 features onboard data logging with a memory capacity of 150,000 datasets and can be deployed for up to 180 days. The device comes equipped with a range of standard sensors and offers customization options for additional sensors. Standard parameters measured by the AS-2000 include DO, specific electrical conductivity, absolute EC, pH, oxidation-reduction potential (ORP), total dissolved solids, resistivity, salinity, seawater specific gravity, and temperature. The AS-2000 also features two auxiliary sockets for additional sensors—one Ion Selective Electrode (ISE) socket and one optical electrode socket—enhancing its versatility for comprehensive water quality monitoring.

### 2.3. Sensor Setup, Calibration, and Validation

Prior to deployment, the AS-2000 multiparameter probe was assembled and configured according to the manufacturer’s guidelines to ensure proper operation and calibration accuracy. All calibration solutions were traceable to ISO and APHA standards and provided by the manufacturer (Aquaread Ltd., Broadstairs, UK). The calibration procedures for each sensor were performed under controlled laboratory conditions (~25 °C) to ensure stable baselines for subsequent in-field measurements of pH, dissolved oxygen (DO), electrical conductivity (EC), turbidity, and nutrients (nitrate and ammonia).

pH calibration: The pH electrode underwent a three-point calibration using standardized buffer solutions at pH 4, 7, and 10, following APHA 4500-H^+^ (2017). Calibration was verified at 25 °C to minimize thermal drift.

DO calibration: The DO electrode was calibrated at both 0% and 100% oxygen saturation, using a sodium sulfite solution for the 0% calibration and a damp-air chamber for the 100% reference point, in accordance with ISO 5814:2012 [29].

EC calibration: The EC sensor was calibrated using the Aquaread RapidCal single-point standard solution, traceable to ISO 7888:1985 [30].

Nitrate and ammonia calibration: The optional ion-selective sensors for nitrate (NO_3_^−^) and ammonia (NH_4_^+^) were calibrated at three concentrations (100 ppm and two 10 ppm solutions). The solutions were temperature-balanced (25 °C ± 1 °C) for the first two calibration points, and the final 10 ppm calibration was conducted at 10 °C lower to assess thermal sensitivity. All calibration standards were pre-diluted and supplied by Aquaread.

During deployment, the probe was inspected weekly to ensure that all electrodes remained submerged and that the protective sleeve was free from sediment buildup, which could otherwise influence readings. The instrument’s automatic temperature compensation maintained measurement consistency under minor thermal fluctuations. The mean river flow rate was recorded to contextualize sensor variability with hydrological conditions.

Validation and Reliability: Although no laboratory grab-sample validation was undertaken during this proof-of-concept phase, all calibration and cross-check procedures followed ISO 7888, ISO 5814, and APHA 4500-H^+^ standards. Previous independent evaluations of the AS-2000 have reported high agreement (R^2^ > 0.95) with laboratory reference methods [31,32].

Future deployments will include systematic cross-validation with laboratory-analyzed water samples using APHA Standard Methods to quantify precision and accuracy under variable hydrological regimes.

### 2.4. Website and Mobile Application Development

A web and mobile application was developed to support monitoring of water quality on the Ystwyth River. The platform provides stakeholders with direct access to live sensor data in a user-friendly format. While technical implementation used modern frameworks (Mapbox API for mapping, Firestore for cloud data management, and Android for mobile accessibility), the design emphasis was based on usability and accessibility for non-specialist users. Android devices were prioritized given their widespread use in rural farming communities and affordability compared to IOS alternatives. The application was structured with simple navigation and large visual markers to make interpretation of results intuitive for farmers and regulators alike.

Key usability considerations included real-time visualization of water quality parameters, historical trend comparison, and automated notifications when critical thresholds were exceeded. These features were selected to minimize the need for specialist interpretation and to facilitate rapid decision-making in agricultural or regulatory contexts.

### 2.5. Sensor Data Collection

Calibration procedures for each sensor on the AS-2000 were established to account for variations in environmental conditions and sensor drift over time. Following calibration, the probe was deployed in the Ystwyth River at the selected site (Figure 1) for continuous data collection over the sampling period. Using the SondeLink V1.07 software provided by Aquaread, data logging intervals were set to 15 min to optimize battery life while maintaining a sufficient level of detail across the data capture period. This setting is appropriate for continuous data collection over extended periods. Prior to deployment, the probe and sensors were tested in situ to confirm that the equipment was functioning correctly and recording data accurately.

These liquid sensors can be affected by fouling and signal drift, which may compromise long-term data quality. To minimize these issues, all sensors used in this study were inspected and recalibrated every two weeks following the manufacturer’s guidelines. This regular calibration schedule helped maintain measurement accuracy and reduce the influence of sensor drift over time. Nevertheless, we acknowledge that extended deployments in highly dynamic environments may still require additional antifouling measures and more frequent maintenance to ensure consistent data reliability.

**Figure 4:**
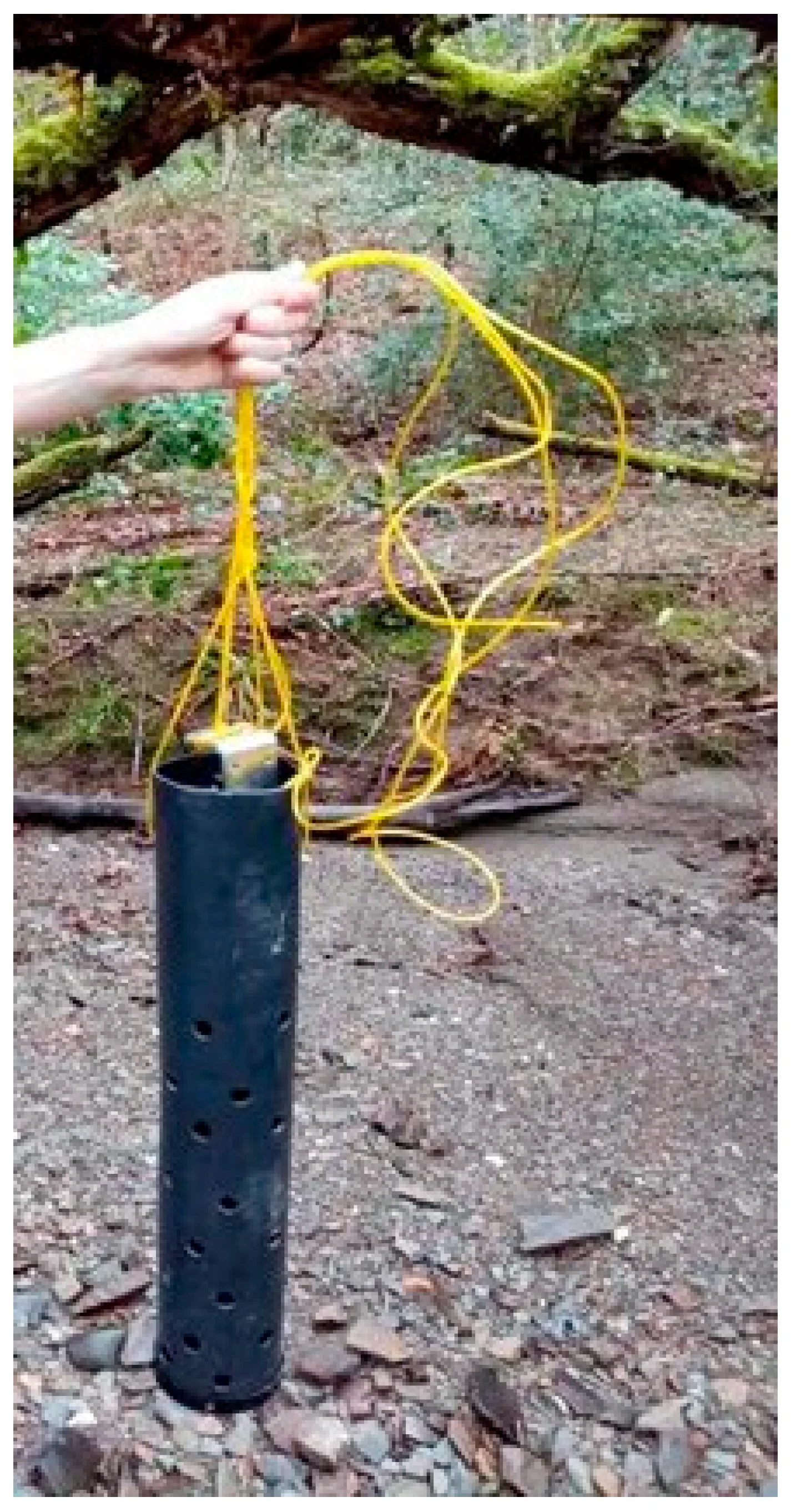
Deployment setup of the AS-2000 within a protective plastic housing that allowed water to flow freely past the sensors while shielding the device from damage due to debris or sediment build-up. The sonde was anchored at a fixed point to ensure consistent submersion and was routinely checked to ensure continued operation and data quality.

The probe was securely installed in the Ystwyth River within a protective plastic housing to minimize the chance of physical damage and sediment build-up, without interfering with the operation of the probe (Figure 4). The probe was anchored in a fixed position with the sensors fully submerged. Regular site visits were conducted to ensure that sediment had not accumulated on the sensors; the probe remained in its correct position, and data was still being recorded.

Data collected by the AS-2000 was stored in its internal memory. On-site manual downloads were performed using a HP ProBook 450 G9 Notebook PC (HP Inc., Bracknell, UK) and the SondeLink V1.07 software. During each download, the probe was temporarily removed from its protective housing and connected to the laptop via a USB cable to transfer the data in CSV format. After the data was successfully downloaded, the probe was returned to its original position in the river to continue data collection.

## 3. Results and Discussion

### 3.1. Web and Mobile Application

A web and mobile application were developed for water quality monitoring in the Ystwyth River, offering dynamic visualization of the key water quality parameters. By providing transparent access to critical data, the application has been designed to empower stakeholders—including farmers, the public, and water-related organization’s—to address environmental pollution risks, optimize resource use, and foster greater agricultural sustainability.

**Figure 5:**
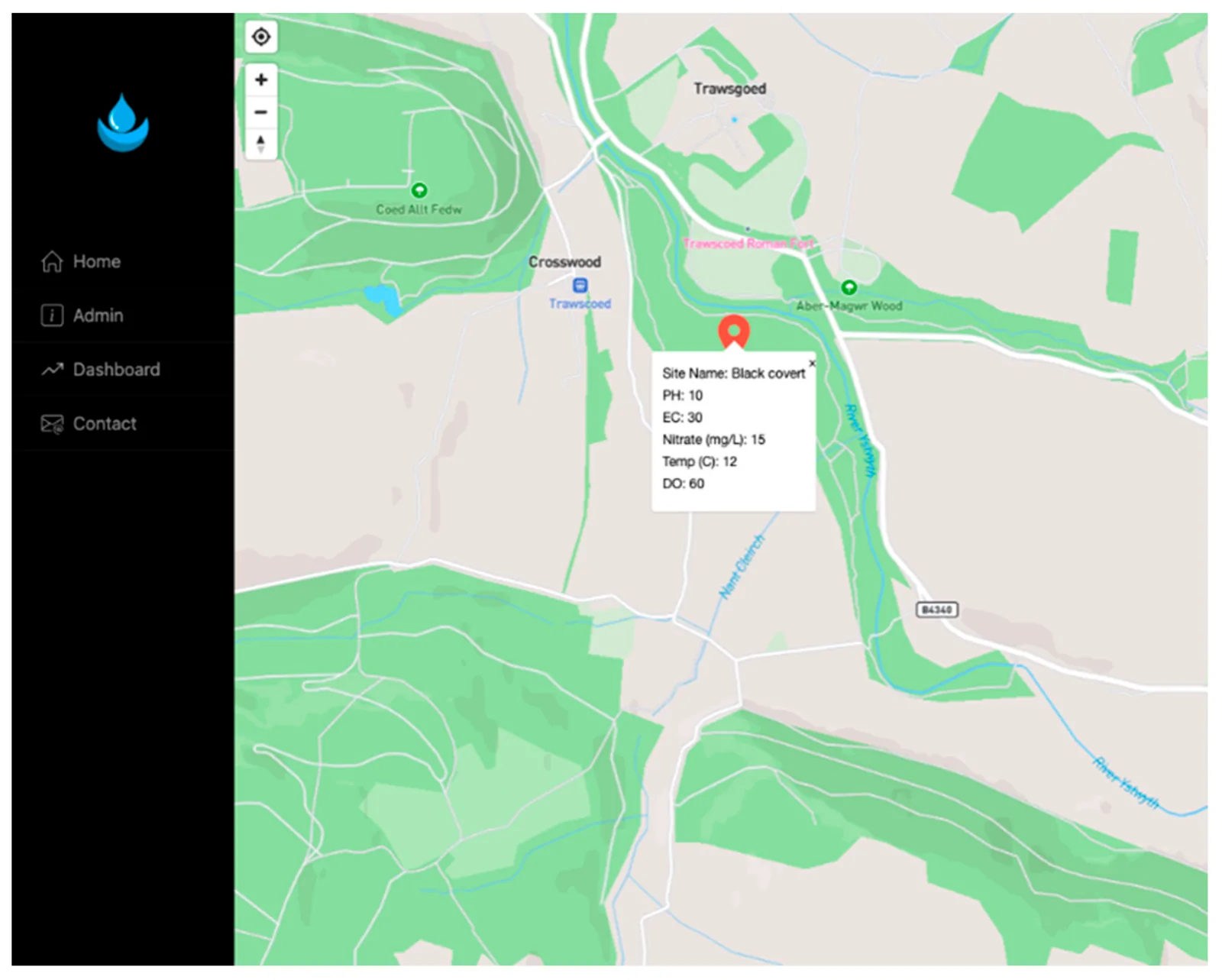
The interactive map interface within the water monitoring application, which displays water quality data from deployed sensors. Each marker corresponds to a monitoring site (the study sample site is shown here). Key water quality parameters are linked to the relevant marker.

**Figure 6:**
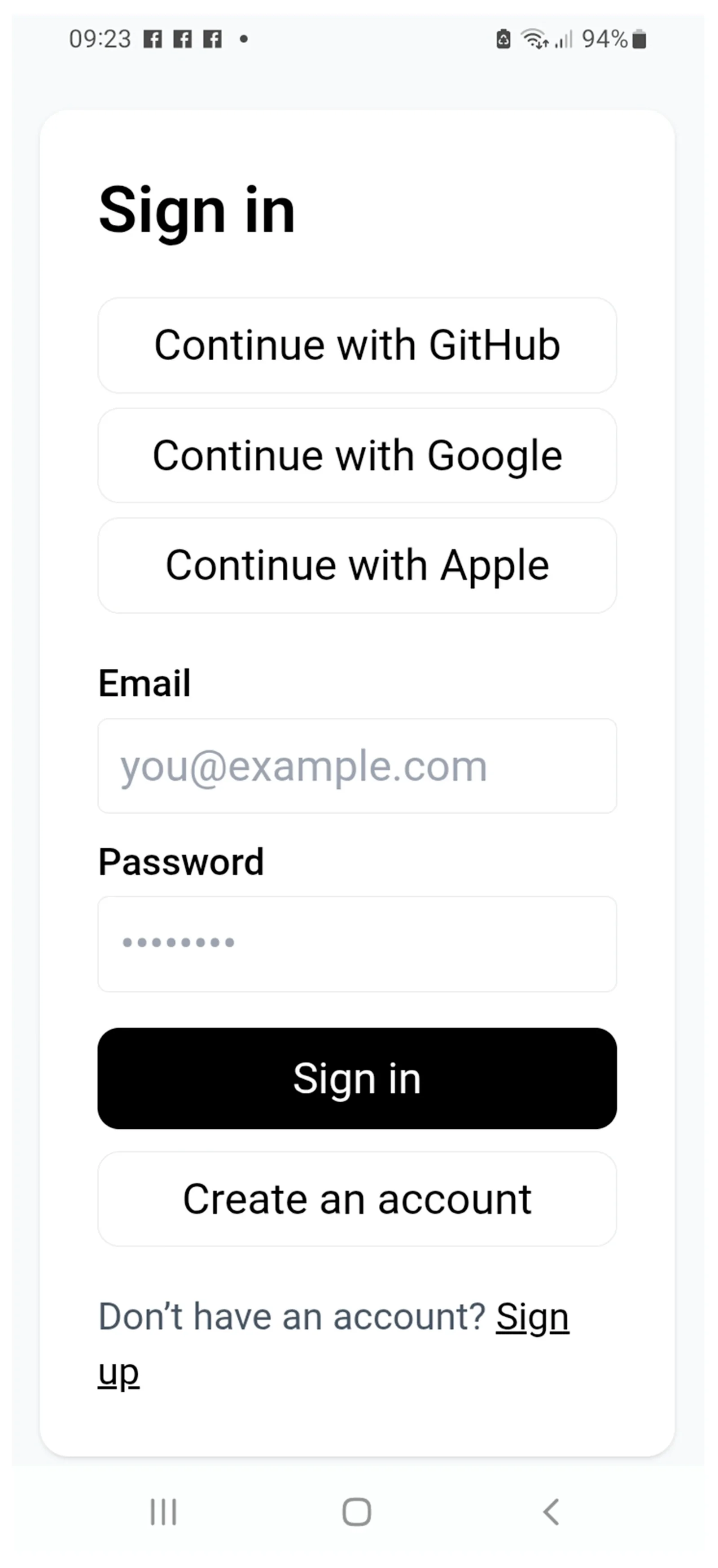
The mobile application interface appears on an Android device, illustrating the log-in page with cross-platform compatibility in a user-friendly format. Users may log in with various third-party accounts, ensuring broad accessibility to the data.

To enhance user engagement and interactivity, the app features an intuitive map interface. As shown in Figure 5, the map view on a tablet displays the precise locations (longitude and latitude) of farms where sensors have been deployed. Each marker represents a water monitoring station along the river. By selecting a marker, users can access detailed data on water quality parameters such as pH, nitrate, ammonia, temperature, and DO levels. Figure 6 demonstrates the mobile application’s cross-platform design on Android devices, ensuring accessibility across various screen sizes and devices commonly used by farmers.

This map-based interface, as illustrated in Figure 5 and Figure 6, allows users to quickly assess the environmental status at specific points along the river, facilitating informed decision-making for both agricultural management and regulatory oversight.

The application leverages Android’s native capabilities, such as GPS location tracking, real-time notifications, and background data synchronization. These features ensure that farmers and environmental stakeholders receive timely alerts about potential water quality issues, even when the app is running in the background.

The practical applications of the system extend beyond technical demonstration. For example, a farmer could monitor nitrate or ammonia spikes following slurry spreading, enabling rapid adjustments to grazing or fertilizer scheduling to reduce runoff risk. Regulators, in turn, could use the application to identify sudden drops in dissolved oxygen or spikes in conductivity that may indicate pollution incidents, enabling faster responses than periodic sampling alone would allow. Early informal demonstrations with academic collaborators and local stakeholders highlighted the value of map-based visualization and automatic alerts as particularly useful for decision-making. While formal usability testing has yet to be conducted, these initial insights underline the system’s potential as a decision-support tool.

### 3.2. Data Analysis Results for Sensor Data

**Figure 7:**
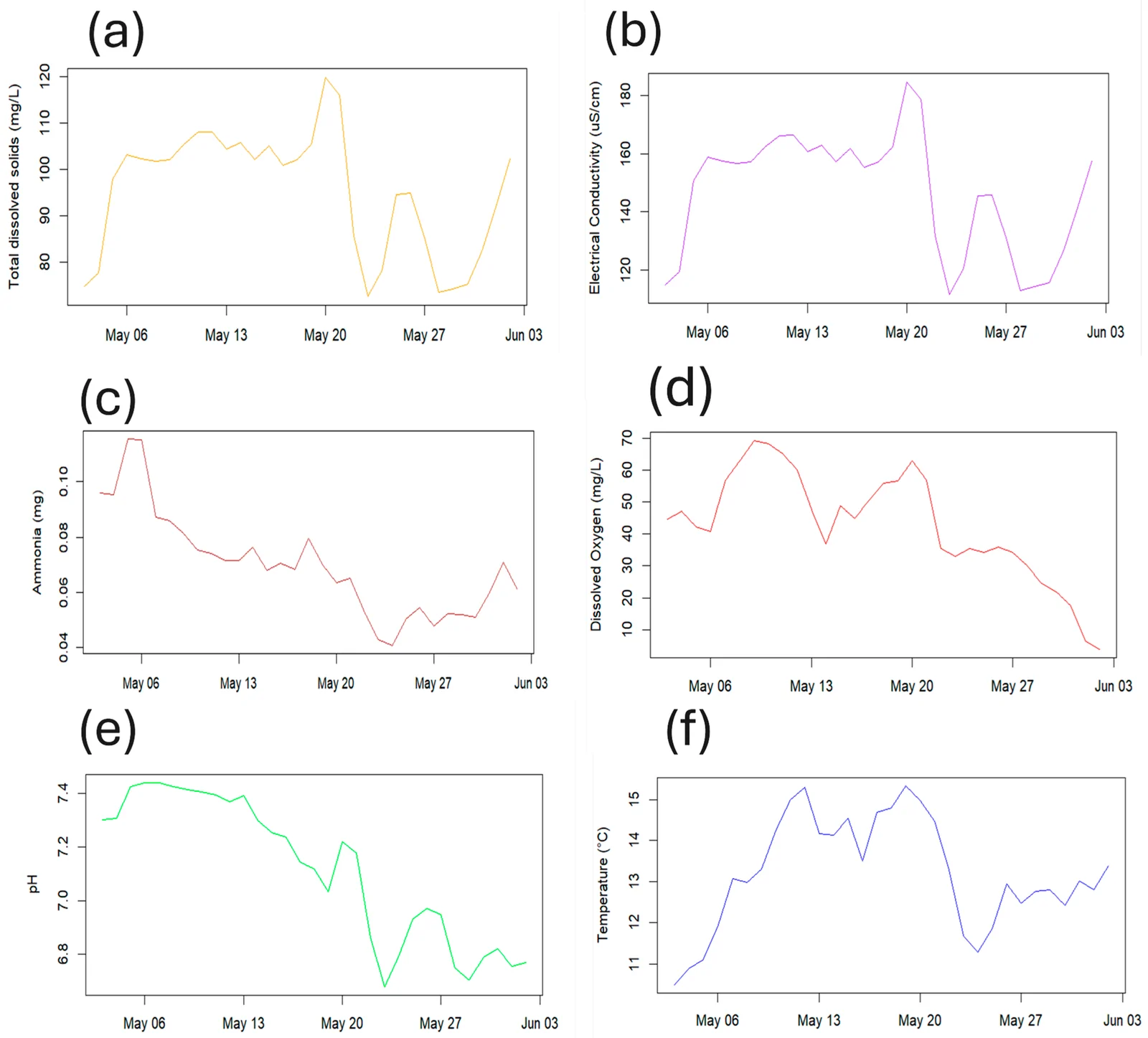

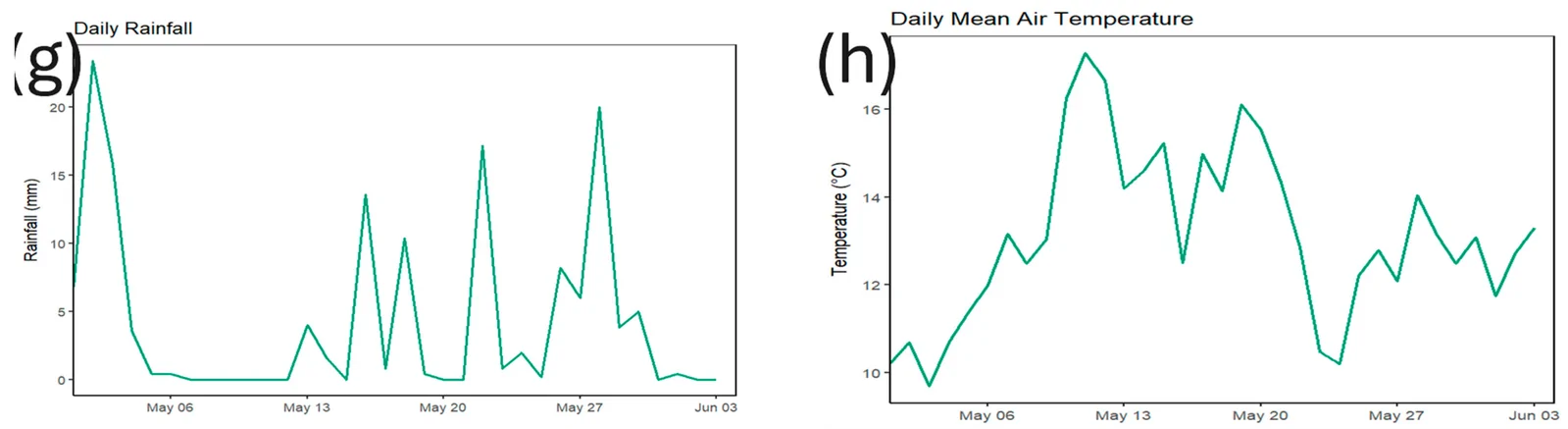
Time-series plots showing variation in environmental parameters during the monitoring period (May–June 2024). (**a**) Total dissolved solids (TDS, mg/L); (**b**) Electrical conductivity (EC, µS/cm); (**c**) Ammonia (mg/L); (**d**) Dissolved oxygen (DO, mg/L); (**e**) pH; (**f**) Water temperature (°C); (**g**) Daily rainfall (mm), with a subtle overlay highlighting rainfall periods; (**h**) Daily mean air temperature (°C).

In addition to the interactive map, the application provides time-series visualizations of critical water quality parameters. Continuous monitoring of the river demonstrated the fluctuations of key water quality parameters over time (Figure 7), showing data for total dissolved solids (TDS), electrical conductivity (EC), ammonia, dissolved oxygen (DO), pH, and temperature. The TDS concentration describes the presence of inorganic salts and minor concentrations of organic substances, which demonstrates a linear relationship with the EC measurement (the capacity of the water to conduct electrical current). A higher proportion of dissolved solids leads to a higher EC, which can arise from agricultural inputs, among other sources. Increased water temperature tends to lead to a higher concentration of ammonia, which can leach into watercourses as a result of agricultural activity and impact water ecosystems. Although nitrate concentrations were measured during this period, results showed negligible levels present. The average river flow rate during the monitoring period was 0.73 m/s, the average atmospheric temperature was 12.84 °C, and the average daily precipitation was 0.33 mm.

Together, these sub-figures illustrate how hydroclimatic drivers (rainfall and air temperature) influenced short-term water-quality dynamics within the Ystwyth catchment, including dilution effects on EC/TDS and temperature-linked variations in DO.

**Figure 8:**
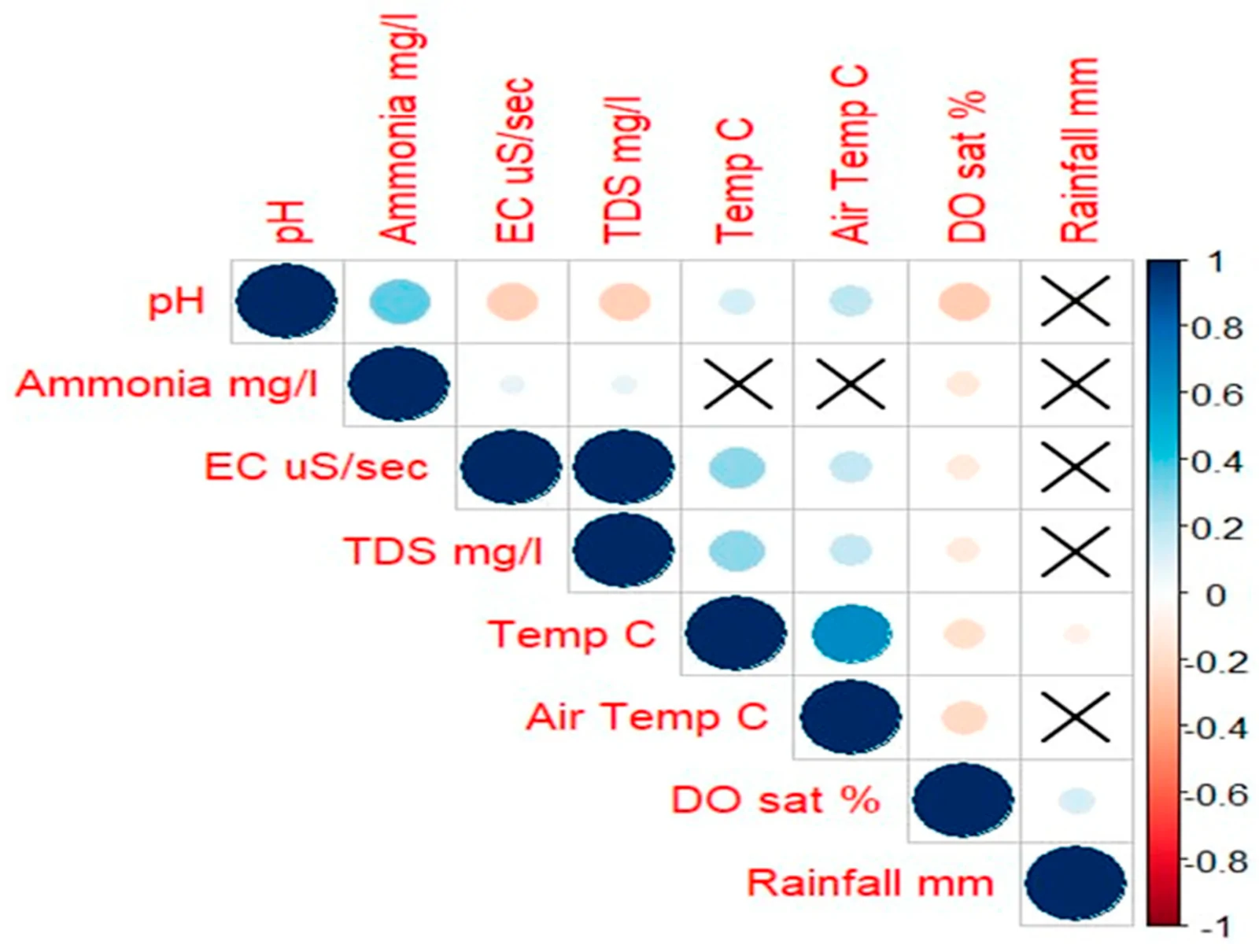
Pearson correlation matrix between environmental parameters. Darker colours indicate stronger correlations between parameters, blue indicating positive correlation and red indicating negative correlations. Crosses indicate non-significant correlations.

Correlations (Figure 8) show that there were strong positive correlations with EC and TDS (correlation coefficient = +1.00, *p* < 0.001) and air temperature with water temperature (correlation coefficient = +0.65, *p* < 0.001). There was a weaker correlation with pH and ammonia concentration (correlation coefficient = +0.36, *p* < 0.001). All other correlations had low likelihoods of having a relationship.

These dynamic plots enable users to visualize fluctuations over the monitoring period and explore temporal variations by scrolling through the data. The ability to identify potential anomalies or outliers, such as sudden drops in DO, is crucial for detecting early signs of river pollution and prompting timely investigations. This dynamic visualization tool, therefore, enhances user engagement and supports real-time analysis, facilitating more informed decisions regarding water quality management.

### 3.3. Linking Environmental Drivers to Sensor Observations

To explore the relationship between environmental conditions and recorded water quality parameters, rainfall and air temperature data were compared qualitatively with the AquaSonde time series. Although the study duration (May–June 2024) was limited, several consistent patterns were observed that indicate causal linkages between environmental drivers and sensor responses. Visually, it can be observed that periods of higher rainfall were consistently followed by declines in electrical conductivity (EC) and total dissolved solids (TDS), reflecting the dilution of solutes and suspended materials by runoff inputs. Conversely, pH and dissolved oxygen (DO) displayed more complex post-rainfall behaviour with a weak negative correlation (correlation coefficient = −0.26, *p* < 0.001), with short-term decreases likely driven by increased organic loading and microbial respiration, followed by gradual recovery during dry periods.

Temperature fluctuations also influenced DO concentrations showing a weak negative correlation (correlation coefficient = −0.20, *p* < 0.001), showing the expected inverse trend where warmer conditions corresponded to slightly lower DO levels. These temporal relationships suggest that the sensor network effectively captures the hydrological and biogeochemical responses of the river system to external climatic and land-use drivers. The observed trends demonstrate the analytical capability of continuous monitoring to reveal dynamic interactions between rainfall events, temperature variation, and nutrient or solute transport processes.

These findings are consistent with known land management patterns in the Ystwyth catchment, where grazing, fertilizer application, and soil disturbance can increase nutrient and organic loads during rainfall-driven runoff events. Future phases of this research will extend the analysis to include quantitative relationships using multi-sensor datasets and longer monitoring periods, allowing for further correlation and regression analyses linking meteorological, hydrological, and land-use parameters to water quality dynamics.

### 3.4. Comparison with Traditional Monitoring Methods

Traditional water-quality monitoring in Welsh rivers typically relies on periodic grab sampling and laboratory analysis, often conducted monthly or quarterly by regulatory agencies such as Natural Resources Wales (NRW). These methods provide high analytical accuracy but are constrained by low temporal resolution, delayed data availability, and higher operational costs due to laboratory processing and field labour. In contrast, the AS-2000 sensor system used in this study offers continuous, high-frequency measurements (every 15 min) that capture short-term variations associated with rainfall, runoff, and diurnal temperature cycles.

The field trial demonstrated that parameters such as electrical conductivity (EC), total dissolved solids (TDS), and dissolved oxygen (DO) responded dynamically to hydrological changes, producing patterns consistent with known biogeochemical processes also documented through traditional monitoring programs. For example, dilution effects following rainfall and oxygen depletion during high organic load events mirror trends typically observed through discrete sampling, confirming that the sensor data are physically meaningful.

While laboratory-based methods remain essential for reference validation and trace metal or nutrient speciation, the real-time sensor approach complements these traditional techniques by enabling early event detection, continuous data streams, and immediate feedback for land and water managers. Integrating both approaches can therefore enhance long-term environmental surveillance and regulatory decision-making within catchments such as the Ystwyth River.

### 3.5. Case Studies Showing That Sensors Capture Short-Term Pollution and Temporal Resolution of Sensor Data

The AquaSonde was deployed for a period of two months (May to end of June) in the Ystwyth River. This has its limitations as water pollution monitoring depends on long-term monitoring of water quality parameters. However, several case studies have shown that sensors capture short-term pollution spikes, which overcomes the limitation of the short deployment time of this study. In one of the case studies [33] in situ sensors were placed in rivers flowing into the Great Barrier Reef in Australia. In this study researchers developed a feature-based framework to detect technical outliers—such as sudden spikes—in turbidity, conductivity, and river level data from in situ river sensors. It successfully identified abrupt changes while keeping false detection rates low. This confirms that such systems can detect short-term anomalies effectively. Another study conducted on the effect of cross-border flow of untreated sewage from Mexico into the USA via the Tijuana River [34]. Researchers evaluated submersible fluorescence-based sensors, measuring tryptophan-like (TRP) and humic-like (CDOM) signals to track water quality in real time. These sensors are especially valuable for detecting rapid changes in organic pollution loads. Work conducted by Rosero-Montalvo et al. (2020) [35] deployed a WSN (Wireless Sensor Network) along Ecuador’s Tahuando River, capturing readings for turbidity, pH, temperature, and other indicators. The system provided real-time data visualization, enabling detection of short-term fluctuations across the river’s course. In a study using Erhai Lake in China, researchers [36] compared 4-hourly data against daily averages to evaluate machine learning model performance (SVR, RF, XGBoost, LSTM) for parameters such as nitrogen, phosphorus, ammonia nitrogen, and dissolved oxygen. Results revealed that high temporal resolution (HTR) data significantly improves accuracy, especially for sequentially dependent variables (e.g., total nitrogen) and models such as LSTM.

### 3.6. Case Studies That Illustrate How Chronic Mine-Derived Pollution Loads Interact with the Shorter-Term Agricultural and Hydrological Fluctuations Captured by the In Situ Sensors Deployed in This Study

In addition to the AquaSonde deployment described here, previous investigations on the Ystwyth catchment have demonstrated the chronic influence of abandoned metal mines, which remain major contributors to Water Framework Directive (WFD) failures. At Frongoch Mine, combined water-quality sampling and flow gauging identified the adits and tailings complex as the largest source of dissolved zinc in the catchment, driving ~32 km of WFD failure downstream [37]. Hydro-geochemical characterization further confirmed zinc concentrations approaching 97 mg/L with limited solubility control, highlighting the requirement for engineered interception and treatment [38]. Monitoring at Cwmystwyth Mine (2009–2012), including MSc-led catchment modelling, identified Pugh’s Adit as the second-largest zinc source in the Ystwyth system [39]. Similarly, Level Fawr Mine contributes over one tonne of harmful metals annually, based on long-term chemistry records and 2013 flow-quality monitoring [40].

These case studies illustrate how chronic mine-derived pollution loads interact with the shorter-term agricultural and hydrological fluctuations captured by the in situ sensors deployed in this study. Together, they demonstrate the importance of coupling continuous real-time monitoring with legacy load assessments: while long-term datasets identify priority sources for remediation, high-resolution sensors capture acute events and compliance risks. This integrated approach supports regulatory objectives under the Water Framework Directive (WFD)and the forthcoming Welsh Sustainable Farming Scheme by linking point-source mine remediation with diffuse agricultural pollution management [41].

### 3.7. Sensor Validation and Limitations

The present deployment focused primarily on demonstrating real-time data acquisition, visualization, and stakeholder usability. As such, the AquaSonde measurements were not cross-validated against laboratory analyses during this phase. However, each probe was calibrated prior to installation following manufacturer’s protocols. This represents a limitation of the current proof-of-concept study. Future work will address this by conducting side-by-side validation campaigns in which sensor readings are compared with laboratory measurements obtained using APHA Standard Methods and spectrophotometric analyses of nitrates, conductivity, and dissolved oxygen. Acknowledging this limitation ensures transparency while setting the foundation for improved data reliability in subsequent deployments.

### 3.8. Policy and Management Relevance

The system developed in this study has direct implications for both agricultural policy compliance and on-farm decision-making. In Wales, regulatory instruments such as the Water Resources (Control of Agricultural Pollution) Regulations 2021 and River Basin Management Plans (RBMPs) under the EU Water Framework Directive require the reduction in nutrient pollution and continuous improvements in river quality [6] By providing real-time measurements of parameters such as nitrate, phosphorus, and dissolved oxygen, this system allows stakeholders to assess water quality against these regulatory standards.

For farmers, the mobile application provides actionable insights into how land management practices—such as fertilizer application, slurry spreading, or livestock grazing—impact local water quality. The ability to identify pollution spikes in real time enables rapid adjustments to farm operations, reducing the risk of non-compliance and potential penalties while supporting the adoption of best practice measures (e.g., riparian buffers, controlled grazing) [7,9]. This decision-support role is particularly relevant in the context of the Sustainable Farming Scheme (planned for 2026), which will incentivize environmentally responsible practices across Wales [15].

For policymakers and regulators, the platform complements existing statutory monitoring networks by offering continuous, high-resolution data streams that are otherwise costly to obtain. This additional evidence base can inform enforcement, support adaptive management strategies at the catchment scale, and improve transparency between regulators, farmers, and the public. In doing so, the system fosters not only compliance with existing legislation but also proactive, collaborative approaches to sustainable water management.

## 4. Future Work

Future work will focus on structured stakeholder engagement to strengthen usability and adoption. Planned activities include pilot deployments with farmers, environmental agencies, and local authorities, supported by scenario-based workshops and iterative feedback sessions. These will guide interface refinements, ensure alignment with farmer practices and regulatory frameworks, and test the effectiveness of communication features such as traffic-light alerts. By embedding continuous feedback loops, the platform aims to evolve into a tool co-designed with end-users, ensuring practical relevance while maintaining its technical robustness.

While this study has demonstrated the feasibility of real-time environmental monitoring for river pollution in Mid-Wales through interactive web and mobile applications, several future directions will expand and enhance the system’s capabilities:

- Pilot deployment and user feedback: The applications developed in this test case have yet to be deployed to farmers and other stakeholders. A pilot deployment phase will focus on introducing the system to end-users (such as farmers, environmental agencies, and local authorities). Information available to farmers for use of the system and in relation to parameter targets will be provided at the time of issue. There will also be a traffic light system incorporated into the app’s design for ease of interpretation of results displayed. Collecting feedback on usability, functionality, and effectiveness will help refine the app’s features and user interface. This iterative approach will ensure the system better meets the needs of those who rely on accurate and timely water quality data for decision-making.
- Large dataset mining and machine learning: To enhance the predictive capabilities of the system, future work will incorporate large dataset mining and machine learning algorithms. By leveraging existing historical data and real-time sensor readings, these techniques can identify patterns and predict potential pollution events. Developing predictive models will enable stakeholders to anticipate water quality issues and take proactive measures before significant pollution occurs, ultimately improving the effectiveness of environmental monitoring.
- Integration with satellite technology: Incorporating satellite data (such as imagery from Landsat) will complement ground-based sensor data by providing a broader spatial context. Satellite-derived water quality metrics, such as turbidity and chlorophyll concentrations, can be compared with on-site sensor readings to enhance monitoring accuracy. This integration will be particularly beneficial for identifying pollution sources across larger catchment areas and remote regions, offering a more comprehensive approach to environmental monitoring.
- Enhanced map features and resource integration: Future iterations of the application will expand map marker functionality to display site-specific pollutant information and provide links to additional resources (such as best practices for pollution mitigation, regulatory guidelines, and educational materials). These enhancements will offer stakeholders context-specific information, fostering a deeper understanding of pollution dynamics and supporting a more informed decision-making process.
- Long-term feasibility of the platform: The Long-term feasibility of the platform will depend on addressing several challenges, including hardware costs of sensors and other water pollution monitoring accessories, ongoing system maintenance, and data security and privacy. Because the platform collects and transmits georeferenced environmental data, ensuring compliance with data protection regulations including the UKGDPR [42] which requires that clear consent mechanisms, data minimization, and transparent user rights and safeguarding sensitive user information are essential. Clear governance structures, anonymization protocols, and transparent data-sharing agreements will be required to build user trust and encourage adoption. It will also be important to clearly define and communicate data governance, ownership, and retention protocols to build user trust and meet regulatory expectations.

### Prioritized Roadmap for Future Development

While the proposed future directions of this study (AI, satellite integration, predictive modelling) highlight important opportunities, a phased roadmap provides clearer priorities:

Short-term (1–2 years):

Pilot deployment and user feedback with farmers, environmental agencies, and local authorities.

- Enhance map marker functionality with pollutant-specific indicators and links to regulatory/best practice resources.
- Establish robust data governance and GDPR compliance protocols to build trust and ensure adoption.
- Medium- to long-term (3–5+ years):
- Develop AI-driven predictive models for forecasting pollution events.
- Integrate satellite observations with sensor data for broader spatial monitoring.
- Scale the platform across Welsh catchments and align it with national water quality frameworks.

This prioritization ensures that near-term actions deliver practical benefits to end-users, while long-term efforts position the system as a scalable, policy-relevant tool for sustainable river basin management.

## 5. Conclusions

This study explored the development and application of interactive mapping and data analysis for environmental monitoring of river pollution, focusing on the Ystwyth River in Mid-Wales. By deploying AquaSonde sensors to measure key water quality indicators, we developed both web and mobile applications that provide an intuitive platform for stakeholders to access real-time sensor readings. This test case demonstrated the feasibility of using real-time monitoring systems to support informed decision-making for water management—particularly regarding pollution risks—while helping to safeguard the environment and enhance agricultural sustainability. Although the web and mobile applications have been successfully developed and tested, the system has not yet been deployed to farmers for field use. Future phases of this project will focus on piloting the app with farmers and other stakeholders to gather feedback on usability, functionality, and effectiveness. This iterative process will help refine the app to better meet the needs of end-users and enhance its impact on water quality management.

Looking ahead, the success of this test case lays the groundwork for scaling up the system to larger catchment areas and broader applications. Future developments will focus on integrating artificial intelligence (AI) to analyze real-time data trends and predict pollution events. AI-driven models can identify patterns, anticipate potential water quality issues, and enable stakeholders to implement proactive measures before significant pollution occurs. Additionally, incorporating satellite technology (such as data from Landsat) will complement ground-based sensors by providing a broader spatial context. This integration will allow users to compare satellite-derived water quality metrics with on-site sensor readings, enhancing monitoring accuracy and coverage.

These advancements hold significant potential for improving environmental surveillance, particularly for farmers and water authorities in remote areas. By providing accessible and transparent water quality data, this system fosters a sense of environmental stewardship among stakeholders. Empowering farmers, policymakers, and community members with real-time insights encourages collective responsibility for maintaining water quality. This approach supports not only regulatory compliance but also a culture of sustainable land and water management, which is essential for protecting aquatic ecosystems and ensuring long-term agricultural viability.

Looking ahead, the success of this test case lays the groundwork for scaling up the system to larger catchment areas and broader applications. As outlined in Section Prioritized Roadmap for Future Development, a phased roadmap will guide this transition: short-term priorities focus on pilot deployment, user feedback, and robust governance, while medium- to long-term developments emphasize predictive modelling, satellite integration, and system scalability. This staged approach ensures that immediate benefits for farmers and regulators are delivered while also positioning the system for long-term policy relevance and environmental impact.

Ultimately, this study highlights both the opportunities and limitations of digital, real-time monitoring platforms in environmental management. By making water quality data accessible and transparent, the system has the potential to foster greater environmental stewardship among farmers, policymakers, and communities. Achieving long-term impact, however, will require balancing innovation with practical considerations of cost, maintenance, and user engagement. If these challenges are addressed, real-time, AI-enabled, and spatially integrated monitoring systems could become powerful tools for promoting sustainable land and water management while protecting aquatic ecosystems.

## Data Availability

Data will be made available on request.

## Abbreviations

CSC	Client-Side Components
CSOs	Combined Sewer Overflows
DO	Dissolved Oxygen
EC	Electrical Conductivity
IDE	Integrated Development Environment
ISE	Ion Selective Electrode
LULC	Land Use and Land Cover
ORP	Oxidation-Reduction Potential
RBMPs	River Basin Management Plans
SEO	Search Engine Optimization
WFD	Water Framework Directive
