# Correction: Akpokodje et al. A Multi-Sensing Technology Approach for the Environmental Monitoring of the Ystwyth River. *Sensors* 2025, *25*, 6743

**DOI:** 10.3390/s26216762

**Published:** 2026-10-26

**Authors:** Edore Akpokodje, Nnamdi Valbosco Ugwuoke, Mari Davies, Syeda Fizzah Jilani, Maria de la Puera Fernández, Lucy Thompson, Elizabeth Hart

**Affiliations:** 1 Department of Computer Science, Aberystwyth University, Aberystwyth SY23 3DB, UK; nnu1@aber.ac.uk; 2 Department of Life Sciences, Aberystwyth University, Aberystwyth SY23 3DA, UK; mwd9@aber.ac.uk (M.D.); elh18@aber.ac.uk (E.H.); 3 Department of Physics, Aberystwyth University, Aberystwyth SY23 3BZ, UK; sfj7@aber.ac.uk; 4 Institute of Biology, Environmental and Rural Sciences, Aberystwyth University, Aberystwyth SY23 3EE, UK; mad79@aber.ac.uk; 5 Department of English and Creative Writing, Aberystwyth University, Aberystwyth SY23 3DY, UK; let22@aber.ac.uk

**References Correction**

There was an error in the original publication [1]. The authors have identified errors in the reference list. Specifically, References 31 and 32 were included incorrectly. The corrected References 31 and 32 appear as follows:

31. Wagner, R.J.; Boulger, R.W., Jr.; Oblinger, C.J.; Smith, B.A. *Guidelines and Standard Procedures for Continuous Water-Quality Monitors: Station Operation, Record Computation, and Data Reporting*; U.S. Geological Survey Techniques and Methods, Book 1, Chapter D3; U.S. Geological Survey: Reston, VA, USA, 2006. https://doi.org/10.3133/tm1D3.
32. Rode, M.; Wade, A.J.; Cohen, M.J.; Hensley, R.T.; Bowes, M.J.; Kirchner, J.W.; Arhonditsis, G.B.; Jordan, P.; Kronvang, B.; Halliday, S.J.; et al. Sensors in the Stream: The High-Frequency Wave of the Present. *Environ. Sci. Technol.* **2016**, *50*, 10297–10307.

The authors state that the scientific conclusions are unaffected. This correction was approved by the Academic Editor. The original publication has also been updated.

## References

[1] Akpokodje E., Ugwuoke N.V., Davies M., Jilani S.F., de la Puera Fernández M., Thompson L., Hart E. (2025). A Multi-Sensing Technology Approach for the Environmental Monitoring of the Ystwyth River. Sensors.

